# Efficacy of a Bariatric Surgery Clinic-Based Pharmacist

**DOI:** 10.1007/s11695-022-06022-y

**Published:** 2022-03-29

**Authors:** Althea Han, Nicole Yvonne Nguyen, Nancy Hung, Salem Kamalay

**Affiliations:** 1 Department of Pharmaceutical Sciences, University of California San Francisco Medical Center, 505 Parnassus Ave, San Francisco, CA 94143 USA; 2 Department of Clinical Pharmacy, University of California San Francisco School of Pharmacy, 513 Parnassus Ave, San Francisco, CA 94143 USA

**Keywords:** Bariatric surgery, Pharmacist, Patient satisfaction, Medication management, Pharmacotherapy

## Abstract

**Purpose:**

To evaluate the impact of a bariatric clinic-based pharmacist on inpatient length of stay, medication errors, and patient experience.

**Materials and Methods:**

This was a retrospective cohort study comparing patients who received a pre-operative pharmacist consultation to historical cases without pre-operative pharmacist consultation prior to admission for bariatric surgery. A patient experience survey was administered post-operatively to the intervention group. The primary outcome was hospital length of stay (LOS). Secondary outcomes included corrected medication errors on reconciliation, pharmacist interventions, adverse drug event (ADE) prevention, and patient satisfaction.

**Results:**

With 68 patients in the intervention group and 67 patients in the control group, the majority were female (76%) and received either laparoscopic Roux-en-Y gastric bypass (53%) or sleeve gastrectomy (47%). The median LOS in the intervention group was 55.5 h, which did not significantly differ from the median 57.9 h in the control group (*p* = 0.56). The clinic-based pharmacist made an average of 13 interventions per patient. Surveys were distributed to 73 patients with a 60% response rate. High overall satisfaction with the pre-operative pharmacist consultation was reported by 97% of patients.

**Conclusion:**

Although hospital LOS did not significantly differ between groups, pre-operative pharmacist consultation prevented potential ADEs, and provided strong patient satisfaction. Having pharmacists as part of a multidisciplinary approach to bariatric surgery patient care can prevent medication-related adverse events and improve patient satisfaction.

**Graphic Abstract:**

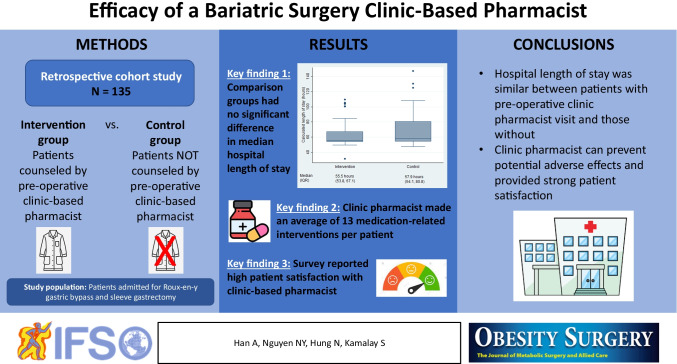

## Introduction

Bariatric surgery can alter the absorption and bioavailability of medications and may increase the risk for potential side effects [1]. Additionally, bariatric surgery candidates often have comorbidities resulting in complex medication regimens that make post-operative medication management difficult. Clinical pharmacists are trained to identify medication-related issues and provide recommendations to reduce potential adverse drug events (ADEs). Inclusion of a pharmacist in medication management in the pre-operative setting adds a layer of safety in preventing medication errors down the line.

Several studies have previously demonstrated that pre-operative medication reconciliation by a pharmacist can reduce medication errors and related adverse events [2, 3]. Pharmacist interventions, defined as recommendations made by a pharmacist, can improve the accuracy of medication lists at the time of hospital admission and better inform patients on specific medication instructions such as holding or stopping medications prior to surgery. Medication reconciliation ensures appropriate medication management through the process of creating an accurate list of all medications that a patient is taking and comparing that list against a provider’s medication orders throughout all of a patient’s transition points within the hospital. Without thorough medication reconciliation in the clinic setting, medication issues are often deferred to the time of admission, when resources and time may be constrained. Additional issues may arise at the time of discharge, requiring urgent and potentially suboptimal medication changes and extra time counseling patients, further delaying time of discharge.

Despite the potential role of pharmacists in bariatric patient management and the value of pre-operative pharmacist interventions, there is currently limited literature available to describe pharmacists’ role in the pre-operative setting for this patient population. For context, our institution is a tertiary medical center based in the USA. The clinical pharmacists at our institution provide recommendations for therapy management to prescribers, and design post-operative medication therapy plans. While the clinical pharmacists do not directly prescribe, as a part of the collaborative care team, licensed prescribers implement the recommendations. The objective of this study is to evaluate the impact of a pre-operative pharmacist consultation in the bariatric surgery clinic at our medical center.

## Material and Methods

### Study Design

In 2018, a clinical pharmacist was integrated into the bariatric surgery clinic to provide medication consultation as part of every patient’s pre-operative clinic evaluation. Each patient was scheduled for a one-time 30- to 60-min meeting with the pharmacist prior to meeting with the surgeon. The clinic pharmacist obtained medication histories and provided recommendations to the patient and the team regarding peri-operative medication management. The pre-operative pharmacist resolved any potential medication-related problems such as those that arise due to medication absorption after bariatric surgery. Additionally, the pre-operative pharmacist provided medication education to the patient.

This retrospective cohort study compared the hospital length of stay (LOS) between patients counseled by a pre-operative pharmacist and those who did not. These represent the intervention and control groups, respectively. Secondary measures included corrected medication errors on reconciliation, pharmacist interventions, ADE prevention, and patient satisfaction. The number of corrections to the prior-to-admission (PTA) medication list and types of interventions made by the pharmacist, including those involving high-risk medications, was evaluated. High-risk medications were defined as medications deemed by the study team to have a high potential to cause adverse effects after bariatric surgery. For example, insulin can cause hypoglycemia after bariatric surgery if not properly adjusted.

To evaluate patient satisfaction, a 5-item patient experience survey was administered to patients with a pre-operative pharmacist consultation using a 5-point Likert-type response scale (strongly agree, agree, neutral, disagree, strongly disagree). Surveys were distributed in-person, via telephone, and online as a Qualtrics survey form. Study personnel and clinic staff were utilized to consent patients and provide the self-administered survey. Patients were asked how likely they were to agree with the following statements:

1. The clinic pharmacist came up with a clear plan for managing my medications after surgery.
2. The pharmacist was knowledgeable about my medications.
3. The pharmacist answered all of my medication-related questions.
4. After speaking with the clinic pharmacist, I felt prepared to manage my medications after surgery.
5. Overall, I was satisfied with my visit with the clinic pharmacist.

This study was approved by our institutional review board.

### Study Population

For the retrospective cohort study, inclusion criteria were adults that were admitted for primary bariatric surgery between January 2015 and October 2018. We excluded patients with concomitant surgeries other than a cholecystectomy. Historical cases were randomly selected and assessed for eligibility. For the patient experience survey, all patients who had a pre-operative pharmacist consultation were eligible.

### Statistical Analysis

The Wilcoxon rank-sum test was used for the primary analysis of LOS. An estimated total sample size of 126 patients was needed to achieve 80% power to detect a difference in LOS of 0.25 days or 6 hours. Given the fact that these patients followed a bariatric pathway that standardizes discharge on post-operative day (POD) 2, we opted to detect a difference in hours instead of days. A 6-hour difference in LOS was acceptable in significance due to the logic that it would increase the number of patients discharged by noon, which is a goal at our institution to facilitate bed control. We chose to combine procedure types when calculating the sample size because the bariatric pathway implemented during the study period proposed discharge on POD2 regardless of whether a patient underwent Roux-en-Y gastric bypass (RYGB) or sleeve gastrectomy. The chi-squared test was used for categorical data and the unpaired *t*-test or Wilcoxon rank-sum test was used for continuous data as appropriate. Analyses were performed with an a priori significance level of 0.05. All statistical analyses were performed with Stata, version 15.0.

## Results

**Fig. 1:**
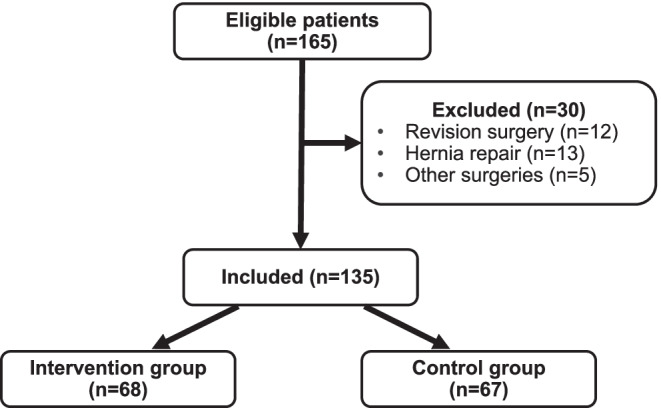
Patient group assignments: all patients meeting inclusion criteria were assigned to either intervention group or control group based on whether or not they received pre-operative pharmacist consultation

Of 165 patients that were assessed for eligibility, 30 had concomitant surgeries and were therefore excluded, leaving 135 patients available for analysis. Of these, 68 patients received pre-operative pharmacist counseling and were categorized as the intervention group. The remaining 65 patients comprised the control group (Fig. 1).

At baseline, the two groups did not have significant differences (Table 1). The study population had a mean age of 48.3 (SD 11.9) and were predominantly female (76%). The two comparison groups were split relatively evenly into two types of bariatric surgeries: Roux-en-Y gastric bypass (53%) or sleeve gastrectomy (47%).

**Table 1:** Baseline characteristics at time of surgery

Measure	Control, *n* = 67	Intervention, *n* = 68
Age (years), mean (SD)	47.7 (12.3)	48.9 (11.6)
Weight (kg), mean (SD)	120.3 (21.4)	122.1 (22.6)
Calculated BMI (kg/m^2^), median (IQR)	41.8 (39.7, 46.8)	41.7 (38.6, 47.4)
ASA category, median (IQR)	3.0 (2.0, 3.0)	3.0 (2.0, 3.0)
Calculated length of surgery (min), median (IQR)	116.0 (92.0, 153.0)	107.5 (81.0, 135.0)
By sleeve gastrectomy	92.0 (76.0, 104.0)	81.0 (73.0, 97.0)
By Roux-en-Y gastric bypass	151.0 (121.0, 180.0)	141.0 (126.0, 163.0)
Laparoscopic, *n* (%)	67 (100%)	68 (100%)
Robot-assisted, *n* (%)	4 (6%)	4 (6%)
Surgeon		
Surgeon A, *n* (%)	24 (36%)	26 (38%)
Surgeon B, *n* (%)	24 (36%)	23 (34%)
Surgeon C, *n* (%)	19 (28%)	19 (28%)
Surgery: sleeve gastrectomy, *n* (%)	34 (51%)	29 (43%)
Roux-en-Y gastric bypass, *n* (%)	33 (49%)	39 (57%)
Discharge day of week: weekday, *n* (%)	35 (52%)	31 (46%)
Year of surgery		
2015, *n* (%)	22 (33%)	0 (0%)
2016, *n* (%)	22 (33%)	0 (0%)
2017, *n* (%)	23 (34%)	0 (0%)
2018, *n* (%)	0 (0%)	68 (100%)
Comorbidities		
Documented infection within 30 days post-op, *n* (%)	9 (13%)	6 (9%)
Diabetes, *n* (%)	23 (34%)	27 (40%)
Hypertension, *n* (%)	36 (54%)	41 (60%)
Sleep apnea, *n* (%)	27 (40%)	35 (51%)
Bleeding disorders, *n* (%)	1 (1%)	1 (1%)
Chronic renal disease, *n* (%)	5 (7%)	5 (7%)
Hemodialysis, *n* (%)	2 (3%)	3 (4%)
Chronic liver disease, *n* (%)	2 (3%)	5 (7%)
Heart failure, *n* (%)	2 (3%)	5 (7%)
MI/stroke/PVD, *n* (%)	1 (1%)	3 (4%)
Chronic pain, *n* (%)	38 (57%)	35 (51%)
Pre-transplant, *n* (%)	2 (3%)	5 (7%)
History of transplant, *n* (%)	4 (6%)	4 (6%)

Abbreviations: *BMI* body mass index, *ASA* American Society of Anesthesiologists, *IQR* interquartile range, *MI* myocardial infarction, *PTA* prior to admission, *PVD* peripheral vascular disease, *SD* standard deviation

**Fig. 2:**
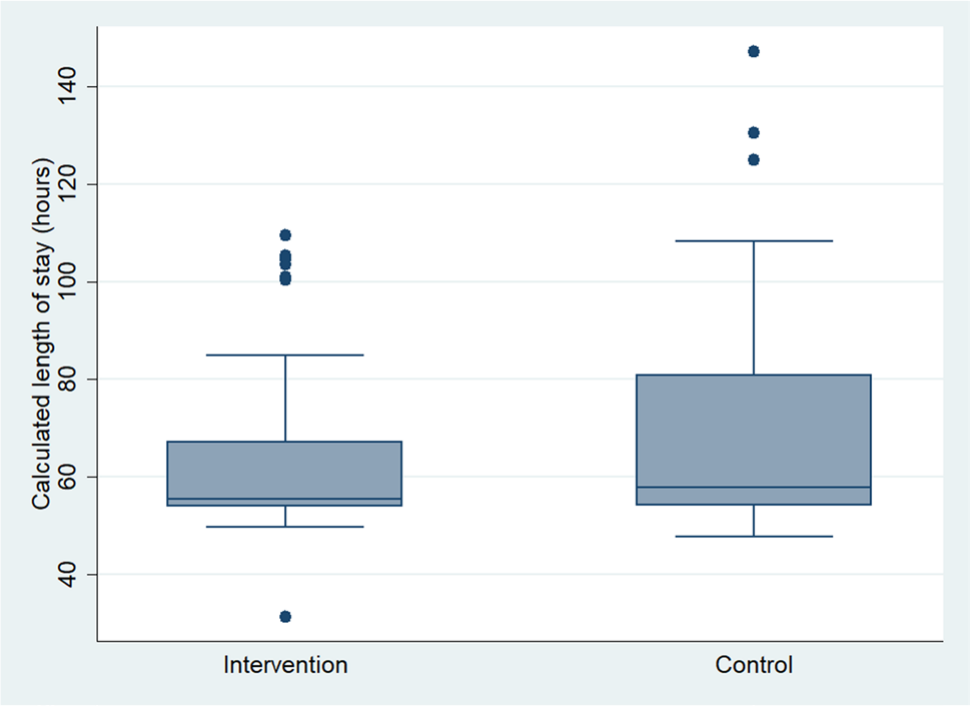
Boxplot of hospital length of stay by group: distribution of hospital length of stay in hours between intervention group and control group

For the primary outcome of hospital LOS, the difference was not statistically significant between the comparison groups (Fig. 2). The intervention group had a median LOS of 55.5 h and the control group had a median of 57.9 h (*p* = 0.56). There was no statistically significant difference in hospital LOS by procedure type.

As part of the medication reconciliation process, corrections to the PTA medication list were made by the clinic pharmacist for 61 patients (90%) in the intervention group. The pharmacist made a median of 4 corrections per patient. Types of corrections included adding missing medications (93%), removing old medications (52%), updating missing or incorrect medication details (48%), and removing duplicate medications (5%).

**Fig. 3:**
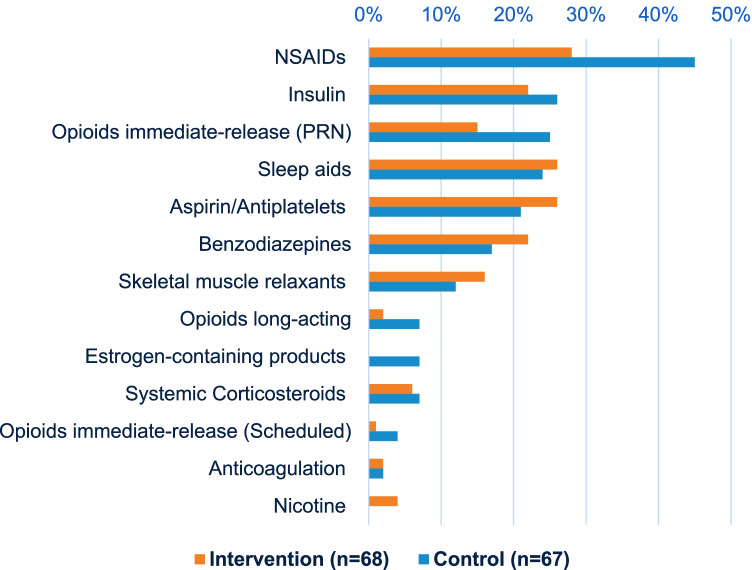
Types of high-risk PTA medications: proportion of historical control cases (in blue) and intervention group (in orange) with high-risk medications listed on their PTA medication list. For the intervention group represented in this figure, high-risk medications are based on updated medication lists after the clinic pharmacist’s medication reconciliation

More of the intervention group reported taking high-risk medications at baseline (73%) compared to the control group (63%). Some of the most common high-risk medications were non-steroidal anti-inflammatory drugs (NSAIDs), insulin, opioids, sleep aids (such as trazodone and Z-hypnotics), aspirin, benzodiazepines, and skeletal muscle relaxants (Fig. 3).

The pharmacist made a median of 13 interventions per patient in the pre-operative clinic consultation. These included recommendations for medication management both before and after surgery (Table 2). Pharmacist interventions for pre-operative management included dose changes, medication tapers, pregnancy prevention counseling, peri-operative anticoagulation planning, and smoking cessation counseling. All patients were counseled on new medications that would be prescribed after surgery.

**Table 2:** Types and total count of pharmacist interventions at time of consultation in the intervention group (*n* = 68)

Pre-operative management	Post-operative management
•Dose change: 11 (16%) •Taper: 9 (13%) •Other interventions^a^: 8 (12%) •Monitoring: 7 (10%)	•New medication counseling: 68 (100%) •Admin instruction change: 58 (85%) •Hold medication until follow-up: 53 (78%) •Agent change: 45 (66%) •Discontinue medication: 41 (60%)

^a^Other interventions: pregnancy prevention counseling (e.g., birth control, barrier method), anticoagulation planning, smoking cessation counseling

Surveys were offered to all patients who received a pre-operative pharmacist consultation, including 5 patients who did not meet inclusion criteria and were not included in the intervention group of the primary analysis. Therefore, a total of 73 patients received the survey. The self-administered patient experience survey response rate was 60%. Survey methods included paper (22), phone (18), and internet survey (4). Over 90% of respondents strongly agreed or agreed to the benefits of an in-clinic pharmacist consultation in the areas of pharmacist clarity, pharmacist answer quality, pharmacist helpfulness, self-preparedness, and overall satisfaction. When asked about overall satisfaction with the pharmacist consultation, 97% of patients reported “strongly agree” or “agree.” Patients’ descriptive comments regarding the pre-operative pharmacist consultation were all positive—samples of quoted comments included:

- “Discharge instructions were well laid out which was appreciated”
- “Knowledgeable and thorough consultation”
- “Well prepared pharmacist”
- “Satisfied with care and all questions were answered”
- “Very helpful to have access to answer questions”

## Discussion

### Hospital Length of Stay

Although the primary outcome of hospital LOS was not significantly different between groups, the intervention group had less variability in LOS, as shown in the box plot of Fig. 2. This may be attributed to a more efficient discharge process after implementation of a pre-operative pharmacist consultation. In the intervention group, patients were counseled extensively prior to surgery on how to manage their medications before and after surgery. The medication management plans were established and discussed prior to hospital admission. This helped to minimize the time spent during the hospital stay to address medication-related issues. However, there are various factors that could impact the hospital LOS after bariatric surgery as reported in current literature. Major et al. [4] found risk factors for prolonged LOS (defined as LOS greater than 3 days) among bariatric patients including low oral fluid intake, high volume of intravenous fluids after surgery, and hospital distance from residence. Mahmood et al. [5] reported sleeve gastrectomy procedure type and pre-operative body mass index (BMI) greater than 50 kg/m^2^ were associated with LOS greater than 1 day. Our study did not evaluate the same risk factors as Major et al., and we did not find high BMI or procedure type to be significant contributors to prolonged LOS.

### Medication List Errors

Despite the lack of a significant difference in the primary outcome between comparator groups, there was a large percentage (90%) of patients with medication errors identified and corrected in the pre-operative pharmacist consultation. An example of a key pharmacist intervention is education on the risks of NSAIDs, which is an established cause of peptic ulcer disease and has also been reported to be associated with marginal ulcer disease after bariatric surgery [6, 7]. Lower rates of NSAID use at the time of surgery among patients in the intervention group compared to the control group may reflect the impact of pre-operative pharmacist education on the risk of post-operative NSAID use.

Only one study was found in the current literature describing similar interventions via pre-operative pharmacist consultation. Silverman et al. [8] described 124 pharmacist consultations, 81.5% of which were pre-operative consultations, where pharmacists provided an average of 8 medication recommendations per patient. Our study reported a higher median 13 interventions per patient, which may be explained by differences in patient characteristics. Additionally, Silverman et al. [8] reported 98% of pharmacist recommendations were approved by surgeons. This study did not measure the percentage of pharmacist interventions accepted, but pharmacist recommendations are generally well received based on our experience.

### High-Risk Medications

The clinic pharmacist provided additional benefit to patients through recommendations aimed at minimizing potential ADEs from high-risk medications.

Using benzodiazepine (BZD) tapers as an example, many clinicians elect to discontinue BZDs after bariatric surgery since this population is susceptible to drug-induced respiratory depression. The risk of respiratory depression is further increased considering most post-operative patients utilize opioids for pain control. Discontinuation of BZDs can be particularly concerning for chronic BZD users given the risk of withdrawal if not properly tapered. BZD tapers over a 4- to 6-week period are generally recommended to minimize withdrawal, which require pre-operative planning and management [9]. In our intervention group, the clinic pharmacist identified use early on and provided appropriate taper recommendations prior to surgery.

Estrogen-containing therapies were another example where pharmacists intervened peri-operatively in this study. Exogenous estrogen is a known risk of venous thromboembolism (VTE), which is the leading cause of mortality after bariatric surgery [10]. Additional VTE risk is avoidable: pharmacist intervention can play a crucial role in preventing VTE-related mortality by minimizing peri-operative use of prothrombotic medications like estrogen.

### Patient Experience Survey

Patients in this study reported high satisfaction with the pre-operative pharmacist consultation, which is consistent with surveys regarding similar pharmacy services reported in the literature. In a patient experience survey of patients who received pharmacist-directed anticoagulation services (*n* = 159), Makowski et al. [11] reported significant increase in amount of information, clarity of information, answer quality, and overall satisfaction compared to those who were managed mainly by their primary care team (*n* = 528). Graham et al. [12] similarly reported high patient satisfaction with pre-operative pharmacist consultation prior to bariatric surgery (*n* = 40), where patients identified pharmacists as particularly helpful in preparing them for medication changes after surgery.

### Limitations of the Study

This study’s power estimation for the primary outcome of hospital LOS was not based on data reported in the literature due to a wide range of LOS reported after bariatric surgery. The heterogenic reporting of LOS may be in part due to differences in surgeon practices and protocols at each bariatric center and advances in practices over time. The time period reviewed in this study was thought to be close enough that practices did not significantly differ. However, a larger sample size may be needed to evaluate the effect of pharmacist intervention on hospital LOS.

Another limitation of this study is that while we were able to characterize medication list corrections and interventions by the pre-operative clinic pharmacist, we were not able to compare this to medication list corrections and interventions made in the control group during hospitalization. We were therefore unable to conclude the degree to which the clinic pharmacist’s contributions were unique and unreproducible in the inpatient setting. However, as previously outlined under sub-section “High-Risk Medications,” it is evident that having accurate medication reconciliation early in the course of pre-operative planning is critical to addressing and ameliorating certain medication-related problems.

It should be noted that medications deemed as high-risk were based on an internal definition; further studies are needed to evaluate long-term adverse outcomes of such medications to better categorize high-risk medications after bariatric surgery.

Patient satisfaction with the pharmacist consultation was evaluated only in patients who received a pre-operative pharmacist consultation. Without a comparator group, we can only say that patients were content with their pre-operative pharmacist consultation. Future studies comparing patient satisfaction to a control group are needed to ascertain if inclusion of a pre-operative pharmacist consultation made a relative improvement in patient satisfaction.

## Conclusions

In conclusion, although the difference in hospital LOS was not statistically significant between patients who received a pre-operative pharmacist consultation and controls, this study demonstrated the value of a pre-operative pharmacist consultation. These benefits included preventing potential ADEs and providing strong patient satisfaction. Future directions to consider include evaluating the risk of continuing high-risk medications after bariatric surgery and pharmacoeconomic analysis of pharmacist interventions with hospital-specific accounting measures.

## Declarations

All procedures performed in studies involving human participants were in accordance with the ethical standards of the institutional and/or national research committee and with the 1964 Helsinki declaration and its later amendments or comparable ethical standards. For the retrospective cohort study: for this type of study, formal consent is not required. For the patient experience survey: informed consent was obtained from all individual participants included in the study.
